# Intimate partner violence and its correlates in middle-aged and older adults during the COVID-19 pandemic: A multi-country secondary analysis

**DOI:** 10.1371/journal.pgph.0002500

**Published:** 2024-05-16

**Authors:** Gwendolyn Chang, Joseph D. Tucker, Kate Walker, Claire Chu, Naomi Miall, Rayner K. J. Tan, Dan Wu

**Affiliations:** 1 Department of Health Services Research and Policy, Faculty of Public Health and Policy, London School of Hygiene and Tropical Medicine, London, United Kingdom; 2 Centre for Population Research in Sexual Health and HIV, University College London, London, United Kingdom; 3 Institute of Global Health and Infectious Diseases, University of North Carolina at Chapel Hill, Chapel Hill, NC, United States of America; 4 Department of Clinical Research, Faculty of Infectious and Tropical Diseases, London School of Hygiene and Tropical Medicine, London, United Kingdom; 5 Gillings School of Global Public Health, University of North Carolina at Chapel Hill, Chapel Hill, North Carolina, United States of America; 6 University of North Carolina Project-China, Guangzhou, China; 7 Saw Swee Hock School of Public Health, National University of Singapore and National University Health System, Singapore, Singapore; 8 Department of Social Medicine and Health Education, School of Public Health, Nanjing Medical University, Nanjing, China; University of Washington Department of Global Health, UNITED STATES

## Abstract

Intimate partner violence (IPV) may have been exacerbated during the COVID-19 pandemic. Middle-aged and older adults, ages 45 years or older, are at higher risk of COVID-19 mortality and social isolation. However, most studies on IPV during the pandemic do not focus on this important subpopulation. Informed by the social-ecological theory, this study examines individual, household, community, and country-level correlates of IPV among middle-aged and older adults in multiple countries using a cross-sectional online survey. Data from 2867 participants aged 45 or older in the International Sexual Health and Reproductive Health (I-SHARE) survey from July 2020 to February 2021 were described using univariate analysis. IPV was defined using four validated WHO measures. Individual characteristics included self-isolation and food security. At the country-level, we examined social distancing stringency. Logistic regression models with a random intercept for country were conducted to explore IPV correlates among 1730 eligible individuals from 20 countries with complete data. Most participants were heterosexual (2469/2867), cisgender (2531/2867) females (1589/2867) between the ages of 45–54 (1539/2867). 12.1% (346/2867) of participants experienced IPV during social distancing measures. After adjustment, participants who self-isolated experienced 1.4 (95% CI 1.0, 2.0, p = 0.04) times the odds of IPV compared to those who had not isolated. Those who reported an increase in food insecurity compared to pre-pandemic experienced 2.2 times the odds (95% CI 1.6, 3.0, p<0.0001) of IPV compared to those who did not report increased food insecurity. People in countries with more stringent social distancing policies were less likely to experience IPV compared to people in countries with lower levels of stringency (aOR = 0.6, 95% CI 0.4, 0.9, p = 0.04). IPV was common among middle-aged and older adults during the COVID-19 pandemic. Our data suggest the need for further crisis management and social protection measures for middle-aged and older adults who have intersecting vulnerabilities to IPV to mitigate COVID-19 impact.

## Background

### IPV among middle-age and older adults

Intimate partner violence (IPV) is defined by the World Health Organization (WHO) as “any behaviour within an intimate relationship that causes physical, psychological, or sexual harm to those in the relationship” by current or former partners [1, 2]. IPV is a pervasive public health issue with an estimated global lifetime prevalence of 30% among women and 10% among men [3, 4]. IPV is also a growing problem that intersects with population aging [5]. Middle-aged and older people are at higher risk of disability, social isolation, chronic illness, and cognitive impairment, which make them more vulnerable to new or worsening forms of IPV [6]. A nationally representative survey conducted in the United States in 2009 of over 5,000 older adults, aged 45 or older, found nearly 10% of older adults faced some form of violence or abuse and that partners and spouses were the perpetrators of more than half of the reported violence [7]. However, research on IPV continues to focus on younger people [8], leaving a gap in the literature about experiences among middle age and older adults who face unique challenges, such as increasing dependence on partners as caregivers [9].

### IPV during COVID-19

During the COVID-19 pandemic, social distancing measures effectively reduced COVID-19 infections, but there were concerns that widespread social distancing measures could put more people at risk of IPV [5, 10, 11]. Older adults who live in care homes were among the first to experience social distancing policies and there was concern that pandemic related policy responses may have exacerbated some IPV risk factors such as isolation and dependency on others for care [12], while also reducing risk of IPV in other ways such as enforcing distancing measures between survivors and perpetrators [13]. There is limited multi-country data on IPV risk among middle-aged and older adults COVID-19 and more research is needed to understand the unintended consequences of social distancing policies on IPV.

### Social-ecological theory

The social-ecological theory operationalises violence at four levels—individual level factors including biological, sociodemographic, and behavioural characteristics; relationship level factors between intimate partners, family members, and peers; community contexts such as residential areas; and larger country-level factors including social norms, gender inequity, social policies [14]. Violence is a complex interplay between each of these levels.

This multi-level theory has been used to understand different types of violence, including IPV [15]. Understanding factors at each of these levels is important in public health to identify populations at risk and develop interventions and policies that address violence at different levels. The social-ecological theory has been adapted for use in this study to accurately reflect the factors captured at each level. ‘Relationship’ was changed to ‘household’ level and ‘societal’ was changed to ‘country’ level. These levels were conceptualised in the same way as Dahlberg and Krug’s (2006) original levels.

Based on a review of the literature conducted to inform this secondary analysis, previously reported correlates of IPV were operationalised at four levels based on an adapted social-ecological theory [14] (Table 1). These were individual-level sociodemographic and behavioral characteristics; household and community level factors capturing characteristics of intimate partners and residential areas; and country-level societal factors including socioeconomic conditions, gender inequity, social policies [14].

**Table 1:** Potential correlates identified from literature on IPV during COVID-19 organised into the social-ecological theory adapted from Dahlberg and Krug (2006).

Individual level	Age [16–19]^$^^+^ Sex [19–21]^$^^+^ Sexual orientation [16,19,20]^$^^+^ Gender minority [16]^$^^+^ Ethnic minority status [19]^$^^+^ Educational attainment [17,19,22]^$^^+^ Employment status [16,17,23,24]^$^^+^ Isolating at home [25–27]^$^^+^ Income level [16,19,23]^$^^?^ Marital status [17,28]^$^^?^ Mental health (stress, anxiety) [18,21,23,28–32]^$^^?^ Change in employment[12,16,32]^$^^?^ Substance use [17,18,22,27,28,32]^$^^?^ Limited social support [23] Symptoms of COVID-19 [12] Health issues [33]
Household and community level	Cohabitation [20,24,28,33]^$^^+^ Food insecurity [34]^$^^+^ Number of children [19,28,29]^$^^?^ Relationship tension or household stress [16,30]^$^^?^ Partner support [30]^$^^?^ Financial difficulties[18,20,22,27,29,33,35] Unstable housing [16,32] Physical conflict [29] Residential area (urban/rural) [16,17,23]^$^^+^ Limited access to resources or healthcare [23,27,32] Housing type [19]
Country level	Country social distancing stringency [28]^$^^+^ Gender inequality [36]^$^^+^ Country of residence [35]^$^^+^ Economic crisis [33]

^$^ collected in I-SHARE 2020–21 or publicly available through country-level indexes.

^+^ included in the final model (except for ethnic minority status due to high missingness).

^?^ conceptualised as mediators and therefore not included in the final model.

### Aim

This secondary analysis seeks to address the gap in literature by analysing correlates of IPV during COVID-19 in middle-aged and older adults at all levels of the social-ecological theory using the International Sexual Health and Reproductive Health in the times of COVID-19 (I-SHARE 2020–21) survey data. The I-SHARE 2020–21 survey is a multi-country online survey that harmonises sexual and reproductive health instruments for global comparison using an open science approach [37].

## Methods

The overall aim of this secondary analysis was to investigate IPV in middle-aged and older adults using a subset of data from the International Sexual Health and Reproductive Health (I-SHARE) conducted during COVID-19 and is the first to do so. Secondary analysis, the use of existing research data to address a question that was different from the original work, is increasingly used in health science [38]. This secondary analysis uses multivariable logistic regression to examine the binary outcome of IPV with multiple categorical correlates–these methods are described in the ‘Secondary Analysis’ sub-section below. Cross-sectional, country-level data using website data verified by academic or international health organizations were collected along with the survey.

We adapted a social-ecological theory to reflect the factors captured at the individual, relationship/household, community, and country levels [14] (Table 1).

### Study design (I-SHARE)

The I-SHARE survey was a cross-sectional online survey administered in 30 low-, middle-, and high-income countries between July 2020 and February 2021 [20]. I-SHARE partnered with national family planning, academic, and non-profit groups in each country as well as global partners like the United Nation Family Planning Association, to support the survey implementation and establish trust in the research [37]. Participants of the I-SHARE survey were at least 18 years old, a current resident of the country where they completed the survey, and able to provide online informed consent [37]. Participants could stop participating or skip any questions they wished. No identifiable data was collected. Country studies linked survivors to local IPV resources.

Each in-country lead researcher organised translation to local languages, field testing, and ethical review [37]. Surveys were piloted for translation on sensitive topics. The I-SHARE survey was distributed in each country through local partner organisations, sexual and reproductive health networks, email listservs, and social media groups determined by the in-country research lead [37]. Due to varying COVID-19 restriction measures, different countries adopted sampling methods that were most contextually feasible at the time being. Twenty-three countries used convenience sampling by distributing the survey through social media, email listservs, sexual and reproductive health networks, and other non-profit and academic partners identified by in-country researchers. Six countries used online panel sampling based on key sociodemographic characteristics identified by in-country researchers and had varying degrees of population representativeness [37]. Two countries used population-representative samples with frames identified by in-country researchers (S1 Table). All surveys were conducted online on personal devices.

The survey captured information on sociodemographic, relationships, sexual health and behaviour, intimate partner violence, and mental health during COVID-19 social distancing measures. Survey questions used a mix of existing validated and newly developed scales [37]. To define the time period of interest for survey items which ask participants about their experiences “during social distancing measures,” I-SHARE researchers determined the start date of social distancing measures in each country based on local policies. Less than 1% of people had been socially distancing for 3 months or less at the time of completing the survey. About 20% had been socially distancing for 3 to 6 months, over 56% for 6 to 9 months, about 15% for 9 months to 12 months, and 10% for over 12 months. Each country survey underwent one to three rounds of testing [37].

### Variables selection and management

Details on the I-SHARE 2020–21 survey are available in S3 Table. Variables were selected from each level of the social-ecological theory for analysis. Variables and confounders identified in the literature (Table 1) were considered potential correlates in this study. Several variables were recoded for analysis to reduce data sparsity issues. These variables are described below:

#### Individual level

Age was collected in the data as a continuous variable but was recoded into age groups, capturing people who are 45–54, 55–64, and 65 or more years old. Sex assigned at birth was collected as ‘male’, ‘female’, or ‘other’. Gender was captured as ‘cisgender,’ ‘non-cisgender,’ and ‘other.’ Those who selected ‘other’ (<1%) were regrouped with ‘non-cisgender.’ Sexual orientation was collected as a categorical variable of ‘heterosexual’, ‘bisexual’, ‘gay’, ‘lesbian’, ‘questioning or unsure’, ‘asexual’, ‘pansexual’, and ‘other’. Very few respondents identified as a sexual minority, so it was recoded as ‘heterosexual’ and ‘other sexual orientation.’ Education level was collected as a categorical variable and recoded based on the UNESCO International Standard Classification of Education (ISCED) categories [48]. Employment status was recoded as ‘unemployed’, ‘employed’ which included those who were self-employed or informal workers, ‘retired’, and ‘other’ which included those who were students (<1%). Isolation due to COVID-19 was captured as a binary ‘yes’ or ‘no’ response. Ethnic minority status was recoded as a binary outcome by the I-SHARE team based on the in-country demographics of the participant.

#### Household and community level

Cohabitation was captured as part of the responses to a question regarding cohabitation and relationship status and recoded to a binary response to only reflect the responses regarding cohabitation. Food insecurity during COVID-19 was recoded to be a binary response: ‘Yes, more than before’ or ‘No or less than before’. At the community level, residential area was captured as a binary ‘urban’ or ‘rural’ response.

#### Country level

At the country level, social distancing stringency was created by categorising the Oxford COVID-19 Government Response Tracker (Ox-CGRT) score of each country as ‘low’ if the score was less than 50 and ‘high’ if the score was greater than 50 [39]. The cut off was determined based on the median stringency score. The measure of COVID-19 social distancing stringency used in this analysis is the mean stringency that the country experienced in the duration of time from when the country’s social distancing measures began to when the surveys were administered. The start of country social distancing measures was determined by I-SHARE in-country researchers.

Gender Inequality Index (GII) scores were recoded as a binary outcome with scores less than or equal to 0.25 as high gender equality and scores from 0.25–0.5 as low gender equality. The cut off was selected based on the median. The most recent GII scores from 2019 represent the gender inequality of each country in the data.

The 2020 Social Progress Index (SPI) was chosen to represent social and environmental protection progress for each country in the study. The range of SPI scores was between about 50–100, so social progress was recoded as a binary with scores less than or equal to 75 as ‘low progress’ and scores higher than 75 as ‘high progress.’ The cut off was selected based on the median.

Country income was based on World Bank 2019–20 criteria. Low- (<$1,085) or lower-middle income ($1,086–4,255) economies were Nigeria, Lebanon, and Mozambique. Upper-middle income ($4,256–13,205) economies were Argentina, Botswana, Colombia, Mexico, Moldova, and Malaysia. High-income (>$13,205) economies were Australia, Canada, Czech Republic, Denmark, France, Germany, Italy, Latvia, Luxembourg, Panama, Portugal, Singapore, Spain, Uruguay, and the United States.

### Outcome: Intimate partner violence

The I-SHARE survey measured physical, sexual, psychological, and financial IPV during COVID-19 social distancing measures using six questions. Of the six questions, five were operationalised in this study to construct psychological, sexual, and physical violence experienced during the COVID-19 social distancing measures, in congruence with the WHO validated Violence Against Women Instrument (VAWI) [40] (S2 Table). Cross sectional studies in both Sweden and Brazil have found that the VAWI demonstrated good construct validity, internal reliability, and ability to differentiate between psychological, physical, and sexual violence within their respective countries [41, 42].

Physical violence was captured from the question, ‘Has a partner slapped, pushed, hit, kicked or choked you or thrown something at you that could hurt you?’ Sexual violence was a composite of responses to two questions which captured forced sexual intercourse and sexual coercion: ‘Has a partner physically forced you to have sexual intercourse when you did not want to?’ and ‘Has a partner made you have sexual intercourse when you did not want to because you were afraid of what your partner might do?’ Psychological violence was a composite of responses to two questions which captured controlling behaviours and emotional abuse: ‘Has a partner tried to restrict (online or phone) contact with your family?’ and ‘Has a partner insulted you or made you feel bad about yourself?’ Financial violence was excluded because it was not part of the validated WHO instrument [43]. Participants could choose between the responses “No,” “Yes, once,” or “Yes, multiple times” for all the violence questions.

A composite IPV variable was created for people who answered all five questions regarding physical, sexual, or psychological violence during the COVID-19 social distancing measures, from July 2020 to February 2021. If people answered ‘yes’ to any of the questions, they were coded as having experienced IPV during the COVID-19 social distancing measures.

### Data inclusion

All data management and analysis were performed using STATA/SE 17.0. Initial exploratory univariate and bivariate analysis with the outcome was conducted for all individual, household, and country-level variables described above. The prevalence of overall IPV and subtypes of IPV was tabulated with univariate analysis. Country social distancing stringency, gender inequality, social progress, and country income level were explored using univariate analysis to describe country level characteristics. We compared the characteristics of those missing data on any of the IPV variables to that of the whole sample and found them to be broadly similar (S4 and S5 Tables).

Of the 23067 participants in the I-SHARE survey, 4454 people were ≥ 45 years old. Participants who had missing data for key variables—age, sex at birth, sexual orientation, relationship status, education level, and geographic area, and any of the five questions used to construct IPV outcome (S2 Table) were excluded from this analysis.

Twenty-four countries were represented in this study—Argentina, Australia, Botswana, Canada, Colombia, Czech Republic, Denmark, France, Germany, Italy, Latvia, Lebanon, Luxembourg, Malaysia, Mexico, Moldova, Mozambique, Nigeria, Panama, Portugal, Singapore, Spain, Uruguay, and United States. The final sub-population included in the descriptive analysis (N = 2867) were middle-aged and older adults who had completed the key sociodemographic items and all five IPV variables (S1 Fig).

### Secondary analysis

We conducted descriptive analysis of these data to understand prevalence of IPV among this sub-population and relevant characteristics. Unadjusted odds ratios of IPV among all potential correlates were explored using logistic regression models with a random intercept for country to account for within-country clustering.

To run a fully adjusted model, people with missing data on gender, social isolation, or food insecurity were dropped by STATA due to modelling requirements. 1730 individuals from 20 countries were included for regression modelling (S1 Table). The multivariable logistic regression model included age, sex at birth, gender, sexual orientation, education level, employment status, social isolation, residential area, cohabitation status, food insecurity, country social distancing stringency level, gender inequality, social progress, and country income level were all identified as potential correlates (S1 Fig). All potential correlates were included in the final model to avoid over-fitting. The random-intercept logistic regression model was fitted with Gauss-Hermite quadrature approximation. Collinearity was checked using a backward-deletion strategy by removing correlates individually and assessing whether the confidence intervals of the remaining correlates narrowed in the model [44]. Correlates were only removed from the model if there was evidence of collinearity. No collinearity was found so all correlates were included in the final model. Likelihood ratio tests were performed to obtain a global p-value for each correlate.

Two sensitivity analyses were conducted, first to test if the model produced similar results in middle-aged and older adults in low- and middle-income countries (LMIC), and second with IPV defined by only physical and sexual violence [20].

### Ethical approval

The I-SHARE survey received ethical approval from the UNC Chapel Hill IRB. Each country applied for IRB approval within their country and this was required for all countries. The University of Ghent research ethical review committee approved the plan to use de-identified data from multiple countries. A data sharing agreement was signed by all collaborating institutions. This secondary analysis was approved by the London School of Hygiene & Tropical Medicine Ethics Committee. Participants provided informed consent and no identifying data was collected.

## Results

### Sample characteristics

Data from 2867 middle-aged and older adults in 24 countries were analysed. The majority of participants were college or university educated (63.7%), heterosexual (86.1%), cisgender (88.6%), females (55.4%), and between the ages of 45–54 (53.7%). Three-quarters were employed and two-thirds lived in an urban area. During the pandemic, most people lived with a partner (77.2%), did not isolate due to COVID-19 (84.7%), and did not experience increased food insecurity (82.9%) (Table 2).

**Table 2:** Description of study sample characteristics from I-SHARE 2020–21 (N = 2867 unless otherwise specified).

		N*	% (Column %)	% of IPV (Row %)
**Age group in years**	45–54	1539	53.7	14.6
	55–64	824	28.7	11.5
	≥65	504	17.6	5.6
**Sex at birth**	Male	1278	44.6	11.3
	Female	1589	55.4	12.8
	Other^1^	0.0	0.0	0.0
**Gender** (N = 2857)	Cisgender	2531	88.6	11.7
	Non-cisgender	326	11.4	15.6
**Sexual orientation**	Heterosexual	2469	86.1	12.0
	Other sexual orientation^2^	398	13.9	13.1
**Ethnic minority status**^**3**^ (N = 1509)	Minority	197	13.1	24.4
	Not ethnic minority	1312	86.9	10.1
**Education level**	No formal and primary	70	2.4	10.0
	Secondary	755	26.3	8.0
	College/University	1826	63.7	13.5
	Other^1^	216	7.5	16.2
**Employment status**	Employed	2154	75.1	12.9
	Unemployed	83	2.9	14.5
	Retired	526	18.4	6.1
	Other^1^	104	3.6	25.0
**Residential area**	Rural	944	32.9	8.7
	Urban	1923	67.1	13.8
**Cohabitation status**	Not living with partner	653	22.8	14.6
	Living with partner	2214	77.2	11.4
**Ever isolated due to COVID-19** (N = 2859)	No	2422	84.7	11.4
	Yes	437	15.3	16.5
**Food insecurity during the pandemic** (N = 1741)	No or less than before	1444	82.9	13.5
	Yes more than before	297	17.1	26.9
**Country social distancing stringency** ^**4**^	Low stringency	897	31.3	12.7
	High stringency	1970	68.7	11.9
**Gender inequity index (GII)** ^**5**^	Low equality	913	31.9	18.8
	High equality	1954	68.2	9.0
**Social progress index (SPI)** ^**6**^	Low progress	570	19.9	22.1
	High progress	2297	80.1	9.7
**World Bank country income level** ^**7**^	High	2118	73.9	9.4
	Upper-middle	735	25.6	19.7
	Low or lower-middle	14	0.5	21.4

^*^ Numbers may differ from the total sample size of 2867 due to missing values.

^1”^Other” was a survey response option. Participants were unable to specify further.

^2^ “Other sexual orientation” includes people who responded as bisexual, gay, lesbian, questioning or unsure, asexual, pansexual, and other.

^3^ Individual’s ethnic minority status was identified based on their country demographics.

^4^ Country social distancing stringency levels were based on the Ox-CGRT score. Countries with ≤50 had low stringency and countries with >50 had high stringency.

^5^ GII measures gender inequalities within a society. Scores ≤0.25 had the most gender equality and scores from 0.25–0.5 had low equality.

^6^ SPI measures the social and environmental progress of a country. Scores ≤75 were considered low and >75 was high.

^7^ Countries were grouped based on World Bank 2019–20 criteria.

Half of the participants resided in a country with a high social distancing policy stringency level (68.7%) and the majority were from countries with higher gender equality (68.2%) and greater social progress (80.1%). More participants were in high-income countries compared to low and middle-income countries.

### Prevalence of overall and subtypes of IPV

Among the 2867 participants in the study 12.1% (346/2867) of people experienced some form of IPV during COVID-19 social distancing measures (Table 3). Ethnic minorities (368/1509) reported more than twice the prevalence of IPV compared to people who were not ethnic minorities (152/1509). The proportion of IPV among people who experienced more food insecurity during the pandemic (468/1741) was almost double that among those who did not experience IPV (235/1741). IPV was also lower in countries with high gender equality (258/2867), social progress (278/2867), and income (269/2867). The frequency of IPV appeared to be almost equal between countries with high (341/2867) and low (364/2867) social distancing stringency levels (Table 2).

**Table 3:** The prevalence of overall IPV and subtypes of IPV, including physical, sexual, psychological violence across the age groups from the I-SHARE 2020–21 survey (N = 2867).

		Percentage of all participants in each age group			
Age groups (years)	N	Any violence	Physical violence	Sexual violence	Psychological violence
45–54	1539	14.6	2.5	2.7	13.0
≥55	1328	9.3	0.9	1.4	8.4
**Overall**	**2867**	**12.1**	**1.8**	**2.1**	**10.9**

The prevalence of overall and subtypes of IPV was lower in people aged 55 or older compared to those aged 45–54 years (Table 3). Psychological violence was experienced by 10.9% (312/2867) of participants. It was more prevalent than physical violence (1.8%, 52/2867) and sexual violence (2.1%, 60/2867). Participants in the oldest group (≥55) reported 6 times more psychological violence than sexual violence and 9 times more than physical violence. Among people 45–54 years, psychological violence was experienced 5 times more than physical and sexual violence. (Table 3).

**Fig 1:**
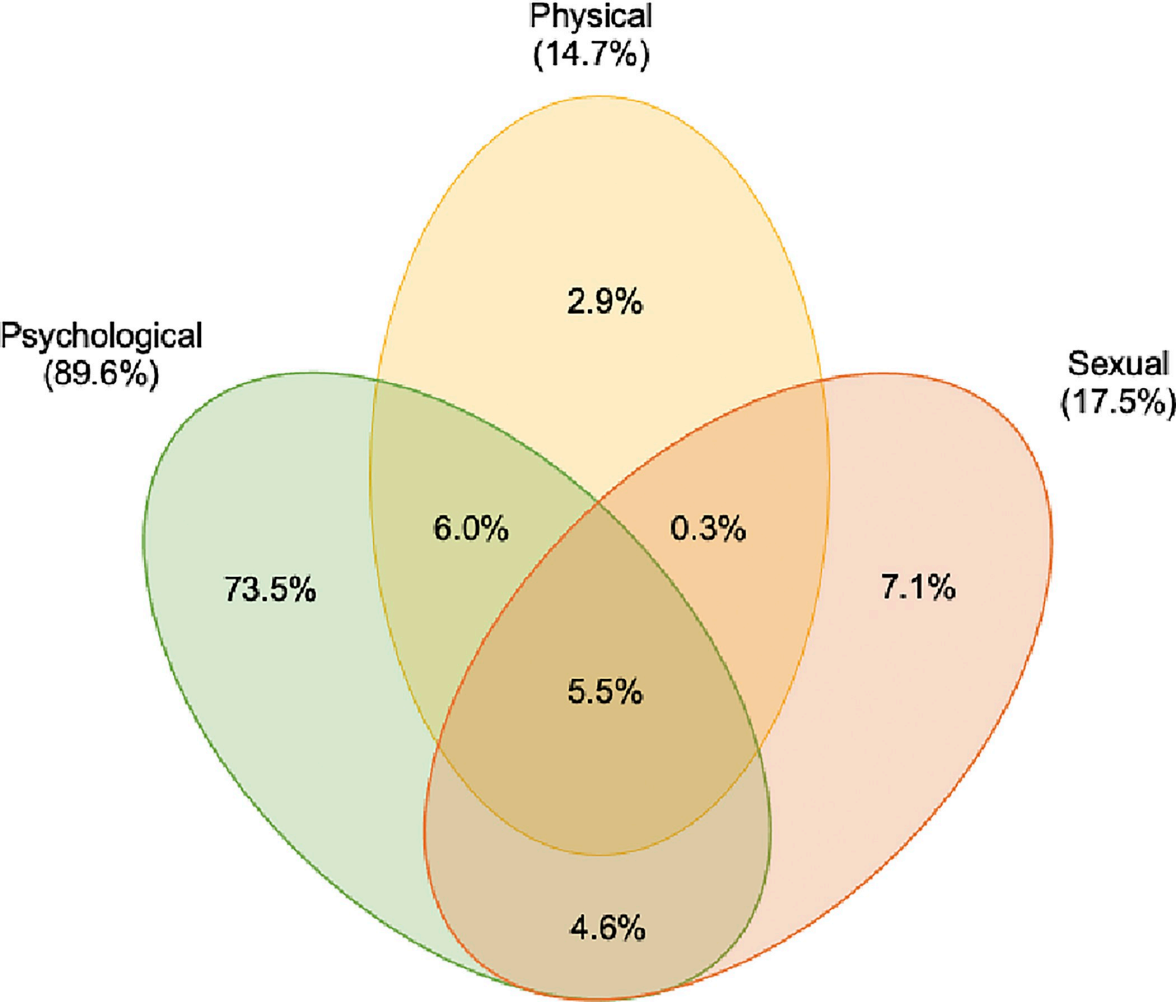
Proportion of each subtype and overlapping subtypes of IPV experienced among middle-aged and older adults during the pandemic in the I-SHARE 2020–21 survey (N = 348). Areas are not proportional to the values. Adapted from Yoshihama and Sorenson (1994).

Among the 348 people who reported experiencing IPV during the COVID-19 pandemic, 16.4% (57/348) experienced two or more forms of violence and 5.5% (19/348) experienced all three subtypes of IPV (Fig 1). 14.7% (51/348) experienced physical violence and 2.9% (10/348) experienced physical violence as their only form of IPV. 17.5% (61/348) reported experiencing sexual violence and 7.1% (25/348) experienced only sexual violence. 89.6% (312/348) experienced psychological violence by their partners and 73.5% (256/348) experienced only psychological violence (Fig 1). Psychological violence was not only the most prevalent subtype of IPV, but also the most frequently co-occurring subtype with the others among middle-aged and older adults who experienced violence during the pandemic. Of the 16.4% (57/348) who experienced multiple forms of violence, 98.2% of them (56/57) experienced psychological violence. 10.4% (36/348) experienced sexual violence and 11.8% (41/348) experienced physical violence.

Physical and sexual violence are often conceptualised as commonly co-occurring with one another. Of those experiencing physical violence, 40% also experienced sexual violence (20/51). Of those experiencing sexual violence, a third also experienced physical violence (20/61).

### Identifying individual, household, community, and country level correlates

Age group, sex at birth, sexual orientation, and education level did not appear to be associated with IPV in bivariate analyses accounting for clustering by country. At the individual level, people who ever isolated due to COVID-19 were more likely to have experienced IPV compared to those who did not after accounting for clustering by country (clustered OR (cOR) = 1.5, 95% CI 1.1, 2.0). At the household level, those who experienced more food insecurity were more likely to have experienced IPV compared to those who did not have increased food insecurity (cOR = 2.4, 95% CI 1.8, 3.3). At the country level, people who lived in high social distancing stringency countries (cOR = 0.5, 95% CI 0.3, 0.8) or highly socially progressive countries (cOR = 0.6, 95% CI 0.4, 0.9) were less likely to have experienced IPV compared to their counterparts.

The final model, which adjusted for all other correlates and accounted for clustering by country, showed that some of the above associations remained significant. Ever being isolated (aOR = 1.4, 95% CI 1.0, 2.0), increased food insecurity during the pandemic (aOR = 2.2, 95% CI 1.6, 3.0), and living in countries with a high level of social distancing stringency (aOR = 0.6, 95% CI 0.4, 0.9) remained significant associated factors. But social progressiveness, gender inequality and country income level were not found to be associated with IPV in the final model (Table 4).

**Table 4:** Unadjusted and adjusted association between correlates and IPV during COVID-19 social distancing among middle-aged and older adults in the I-SHARE 2020–21 survey (N = 1730)^1^.

			Unadjusted OR (95% CI)^2^	Final model: aOR^3^ (95% CI)	Global P-value^4^
**Individual Level**	Age group in years	45–54	1	1	0.54
		55–64	0.8 (0.6, 1.1)	0.9 (0.6, 1.2)	
		≥65	0.7 (0.4, 1.1)	0.7 (0.4, 1.4)	
	Sex at birth	Male	1	1	0.40
		Female	1.0 (0.8, 1.3)	0.9 (0.7, 1.2)	
	Gender	Cisgender	1	1	0.18
		Non-cisgender	1.2 (0.8, 1.9)	1.3 (0.9, 2.0)	
	Sexual orientation	Heterosexual	1	1	0.97
		Other sexual orientation	1.0 (0.7, 1.4)	1.0 (0.7, 1.5)	
	Education level	No formal and primary	1	1	0.89
		Secondary	0.8 (0.3, 2.2)	1.0 (0.4, 2.6)	
		College/University	0.9 (0.3, 2.3)	1.1 (0.4, 2.5)	
		Other	1.0 (0.3, 2.9)	1.2 (0.4, 3.3)	
	Employment status	Employed	1	1	0.03
		Unemployed	1.3 (0.7, 2.5)	1.2 (0.6, 2.4)	
		Retired	0.8 (0.5, 1.4)	1.1 (0.6, 2.0)	
		Other	2.3 (1.4, 3.8)	2.2 (1.3, 3.7)	
	Ever isolated due to COVID-19	No	1	1	0.04
		Yes	1.5 (1.1, 2.0)	1.4 (1.0, 2.0)	
**Household & Community Level**	Cohabitation status	Not living with partner	1	1	0.81
		Living with partner	1.0 (0.7, 1.4)	1.1 (0.8, 1.4)	
	Food insecurity during the pandemic	No or less than before	1	1	<0.0001
		Yes more than before	2.4 (1.8, 3.3)	2.2 (1.6, 3.0)	
	Residential area	Rural	1	1	0.80
		Urban	1.2 (0.8, 1.7)	1.0 (0.7, 1.5)	
**Country Level**	Country social distancing stringency	Low stringency	1	1	0.04
		High stringency	0.5 (0.3, 0.8)	0.6 (0.4, 0.9)	
	Gender inequality index (GII)	Low equality	1	1	0.41
		High equality	0.7 (0.5, 1.1)	0.8 (0.5, 1.3)	
	Social progress index (SPI)	Low progress	1	1	0.14
		High progress	0.6 (0.4, 0.9)	0.4 (0.1, 1.4)	
	World Bank country income level	High	1	1	0.49
		Upper-middle	1.4 (0.8, 2.3)	0.5 (0.2, 1.7)	
		Low or Lower-middle	1.7 (0.4, 6.7)	0.7 (0.1, 4.2)	

^1^ Total N is lower due to missing data.

^2^ Accounts for clustering by country.

^3^ Adjusted for all other covariates. Accounts for clustering by country. The final model includes data from 20 countries—Australia, Canada, Colombia, France Germany, Italy, Latvia, Lebanon, Luxembourg, Malaysia, Mexico, Moldova, Mozambique, Nigeria, Panama, Portugal, Singapore, Spain, Uruguay, and USA.

^3^ This is the p-value for the final model. Global p-values were determined by likelihood ratio tests.

Note: Ethnic minority status was excluded in regression models due to missing values

### Sensitivity analyses

A sensitivity analysis among the 749 participants (S6 Table) from LMIC resulted in the same overall associations between social isolation (aOR 1.8, 95%CI 0.6, 1.7, p = 0.02), food insecurity (aOR 3.4, 95% CI 1.5, 3.8, p = 0.0004), and country social distancing stringency level (aOR 0.5, 95% CI 0.3, 0.8, p = 0.02) (S6 Table). A sensitivity analysis among 2867 participants to operationalise IPV with another standard definition which only includes physical and sexual violence resulted in similar directions and effect sizes for social isolation (aOR 1.6, 95% CI 0.9, 2.8, p = 0.13), food insecurity (aOR 2.0, 95% CI 1.2, 3.5, p = 0.02), and country social distancing stringency level (aOR 0.5, 95% CI 0.3, 1.0, p = 0.06) compared to the final model (S7 Table). However, fewer correlates appeared to be associated with this IPV outcome.

## Discussion

12.1% of middle-aged and older adults reported experiencing IPV in the period between their local social distancing measures and the I-SHARE survey. The prevalence of IPV was the same between males and females and decreased as age group increased. Psychological violence was the most common subtype of violence. Increased isolation, increased food insecurity and lower social distancing stringency were associated with greater IPV among middle-aged and older adults. This secondary analysis of I-SHARE 2020–21 extends the literature by focusing on middle-aged and older adults using multi-country data.

The overall prevalence of IPV during COVID-19 among middle-aged and older adults estimated by this study was found to be higher than the 7.0% (1,070/15,336) of participants from the whole I-SHARE sample who reported experiencing IPV.

The most common form of IPV across all middle and older age groups was psychological violence. This is consistent with the existing literature on IPV during COVID-19 and broader patterns seen in IPV [45–47]. Psychological violence was found to overlap with physical and sexual subtypes of IPV. Previous studies suggest that psychological violence is an important risk factor for other types of violence [45, 46]. Several studies have highlighted the extreme toll that psychological violence has on mental distress [24, 48], self-esteem, feeling worthless, loss of identity, as well as a higher prevalence of post-traumatic stress disorder (PTSD) and complex-PTSD [47, 49–51]. Unfortunately, middle-aged and older people experiencing IPV during the pandemic also had reduced access to IPV support services [52] and general mental health resources [53]. Low digital connectivity among this age group has also made many remote service replacements inaccessible, leaving them without access to necessary support [54].

### Individual level

There was strong evidence that having isolated due to COVID-19 was associated with increased odds of IPV compared to those who did not isolate among middle-aged and older adults. This finding coincides with a broader base of literature that identifies social isolation as a risk factor for IPV [25, 55, 56] and conversely that social support is protective [26]. During the pandemic, many people self-isolated [57]. There are several possible explanations for the observed association between social isolation and experiences of IPV. Isolation leads to weakened social networks, decreasing help-seeking opportunities while increasing a perpetrator’s ability to control and coerce survivors [25].

### Household level

Higher odds of IPV were observed in people who experienced increased food insecurity during the COVID-19 pandemic compared to those who did not. The association between food insecurity and IPV has been well established in many countries [58–60]. Food insecurity is often discussed in the context of a general lack of material security, such as housing or economic insecurity [58–60]. Stress theory suggests that insufficient or limited resources at the individual and household level is a source of tension that can increase risk of IPV [59, 61]. It posits that stress associated with too much demand over too little resources leads to IPV [61].

### Country level

We found lower odds of IPV in countries with higher social distancing stringency. Current literature on the effect of social distancing policies on IPV has produced mixed evidence on its relationship with IPV [10], and in many previous studies, social distancing was found to be associated with an increased prevalence of IPV while simultaneously keeping IPV hidden [5, 16, 21]. Our study findings among middle-aged and older adults were inconsistent with most of these previous findings. One potential explanation may be that these countries with more stringent social distancing policies might also be more likely to implement stronger social protections [62, 63]. Literature on social protection during COVID-19 shows that cash transfers were the most commonly implemented social protections, as well as food vouchers and wage subsidies [63]. These protections were primarily implemented by high-income countries alongside social distancing policies [63]. Without indexes that capture the impact of concurrent COVID-19 policy responses, the relationship between social distancing stringency, social protections, and IPV cannot be disaggregated.

### Strengths and limitations

There were several strengths of this study. Multi-country data from I-SHARE 2020–21 offered an opportunity to examine correlates of IPV among older adults from a large dataset with participants from diverse geographic regions and country attributes. The large number of participants allowed increased generalisability of findings compared to single-country studies. Analysis of country-level variables was a unique strength of this study and produced nuanced insights into how COVID-19 social distancing policies might affect IPV among older adults.

The study also has some limitations. We operationalised IPV with validated measures from the WHO Violence Against Women Instrument (VAWI). Cross sectional studies from Sweden and Brazil have shown that VAWI has construct validity and internal reliability [42, 43]. The I-SHARE study used an adapted version of this validated measure, using five of the six questions from the VAWI. Therefore, in this secondary analysis, we were only able to construct the outcome of IPV with these five questions, which has unknown effects on the construct validity and reliability of the measure. However, our sensitivity analyses showed that the construction of IPV used in this study is robust.

I-SHARE was a cross-sectional online survey and due to this design, we are unable to determine the direction of association between correlates and outcomes. As a result, causal claims cannot be made for any of the correlates identified in this study. Additionally, participants may not be comfortable disclosing experiences of IPV which could lead to an underestimation of the prevalence of IPV.

Missing data from this study could impact the generalisability of the findings and is a limitation. About 30% of people had missing IPV outcome data. However, people with missing data for IPV had similar sociodemographic characteristics to those people who were included in the descriptive analysis. Efforts were made to increase the generalisability of these findings by using diverse sampling methods in the I-SHARE survey, such as including population representative sampling frames and using online panels. Sensitivity analysis showed that the model is also sensitive across LMIC subpopulations, with the same directions of associations and larger effect sizes compared to the final model.

## Conclusion

Our analysis adds to our understanding of some unique factors associated with IPV among middle-age and older adults during COVID emergency, but further studies are needed to understand the pathways through which country-level factors, such as social distancing and social protection policies, might have impacted IPV in various country settings. Intimate partner violence and the intersection of aging is a complex phenomenon that needs to be understood in the context of suites of pandemic and crisis policies, which interact in multifaceted ways to shape experiences of violence. Detailed examination of mediation effects and cross-level interactions within the social-ecological theory is needed to continue to expand the understanding of how correlates are associated with IPV in middle age and older adults.

## Supporting information

S1 Data I-SHARE 2020–21 data used for analysis. (DTA)

S1 Fig Descriptive analysis population (N = 2867) and bivariate and final model population (N = 1730) selection parameters from I-SHARE 2020–21. (DOCX)

S1 File I-SHARE 2020–21 codebook. (XLSX)

S1 Table Number and percentage of countries in I-SHARE 2020–21 by sampling strategy. Low- (<$1,085) or lower-middle income ($1,086–4,255) economies were Nigeria, Lebanon, and Mozambique. Upper-middle income ($4,256–13,205) economies were Argentina, Botswana, Colombia, Mexico, Moldova, and Malaysia. High-income (>$13,205) economies were Australia, Canada, Czech Republic, Denmark, France, Germany, Italy, Latvia, Luxembourg, Panama, Portugal, Singapore, Spain, Uruguay, and the United States.
(DOCX)

S2 Table I-SHARE 2020–21 adaptations of the WHO violence against women instrument (VAWI) questions used to construct IPV. (DOCX)

S3 Table Questions on the I-SHARE 2020–21 survey used for the analysis. (DOCX)

S4 Table Characteristics of people missing and not missing IPV outcome responses in the study from I-SHARE 2020–21 (N = 4057). (DOCX)

S5 Table Characteristics of people excluded from the final model due to missing data on gender, isolation, and food insecurity from I-SHARE 2020–21 (N = 2867). (DOCX)

S6 Table A sensitivity analysis with participants from LMICs to determine if the model is sensitive within this population from the I-SHARE 2020–21 survey (N = 749). (DOCX)

S7 Table Sensitivity analysis of the construction of IPV. It is defined in this analysis as physical and sexual violence using data from the I-SHARE 2020–21 survey (N = 2867).
(DOCX)

## References

[1] World Health Organization. Violence Info: Intimate Partner Violence [Internet]. 2022. Available from: https://apps.who.int/violence-info/intimate-partner-violence/

[2] Garcia-Moreno C, Guedes A, Knerr W. Understanding and addressing violence against women. World Health Organ [Internet]. 2012; Available from: https://www.who.int/publications/i/item/WHO-RHR-12.36

[3] Centers for Disease Control and Prevention. Intimate Partner Violence, Sexual Violence, and Stalking Among Men. 2020; Available from: https://www.cdc.gov/violenceprevention/intimatepartnerviolence/men-ipvsvandstalking.html

[4] World Health Organization. Violence against women prevalence estimates, 2018: global, regional and national prevalence estimates for intimate partner violence against women and global and regional prevalence estimates for non-partner sexual violence against women. 2021; Available from: https://www.who.int/publications/i/item/9789240022256

[5] Emandi R, Encarnacion J, Seck P, Tabaco R. Measuring the Shadow Pandemic: Violence against women during COVID-19. UN Women [Internet]. 2021 Nov 24; Available from: https://data.unwomen.org/publications/vaw-rga

[6] Gerino E, Caldarera AM, Curti L, Brustia P, Rollè L. Intimate Partner Violence in the Golden Age: Systematic Review of Risk and Protective Factors. Front Psychol [Internet]. 2018 [cited 2022 Dec 23];9. Available from: https://www.frontiersin.org/articles/10.3389/fpsyg.2018.01595 30233454 10.3389/fpsyg.2018.01595 PMC6131561

[7] Acierno R, Hernandez-Tejada MA, Anetzberger GJ, Loew D, Muzzy W. The National Elder Mistreatment Study: An 8-year longitudinal study of outcomes. J Elder Abuse Negl. 2017 Aug 8;29(4):254–69. doi: 10.1080/08946566.2017.1365031 28837418

[8] Yakubovich AR, Stöckl H, Murray J, Melendez-Torres GJ, Steinert JI, Glavin CEY, et al. Risk and Protective Factors for Intimate Partner Violence Against Women: Systematic Review and Meta-analyses of Prospective–Longitudinal Studies. Am J Public Health. 2018 Jul;108(7):e1–11. doi: 10.2105/AJPH.2018.304428 29771615 PMC5993370

[9] Pathak N, Dhairyawan R, Tariq S. The experience of intimate partner violence among older women: A narrative review. Maturitas. 2019 Mar 1;121:63–75. doi: 10.1016/j.maturitas.2018.12.011 30704567 PMC6546119

[10] Peterman A, O’Donnel, Palermo T. COVID-19 and Violence against Women and Children.

[11] Guterres A Warning of Rise in Gender-Based Violence during COVID-19 Pandemic, Secretary-General Observance Message Says Policies to Tackle Root Causes, Protect Women Can End Abuse | UN Press [Internet]. [cited 2022 Dec 23]. Available from: https://press.un.org/en/2021/sgsm21034.doc.htm

[12] Davis M, Gilbar O, Padilla-Medina DM. Intimate Partner Violence Victimization and Perpetration Among U.S. Adults During the Earliest Stage of the COVID-19 Pandemic. Violence Vict. 2021 Oct 1;36(5):583–603. doi: 10.1891/VV-D-21-00005 34725264

[13] Campbell L, Tan RKJ, Uhlich M, Francis JM, Mark K, Miall N, et al. Intimate Partner Violence During COVID-19 Restrictions: A Study of 30 Countries From the I-SHARE Consortium. J Interpers Violence. 2023 Jun 1;38(11–12):7115–42. doi: 10.1177/08862605221141865 36703528 PMC9895276

[14] Dahlberg LL, Krug EG. Violence a global public health problem. Ciênc Saúde Coletiva. 2006 Jun;11(2):277–92.

[15] Heise LL. Violence against women: an integrated, ecological framework. Violence Women. 1998 Jun;4(3):262–90. doi: 10.1177/1077801298004003002 12296014

[16] Peitzmeier SM, Fedina L, Ashwell L, Herrenkohl TI, Tolman R. Increases in Intimate Partner Violence During COVID-19: Prevalence and Correlates. J Interpers Violence. 2022 Nov;37(21–22):NP20482–512. doi: 10.1177/08862605211052586 34866451 PMC9014340

[17] Miller AP, Mugamba S, Bulamba RM, Kyasanku E, Nkale J, Nalugoda F, et al. Exploring the impact of COVID-19 on women’s alcohol use, mental health, and experiences of intimate partner violence in Wakiso, Uganda. PloS One. 2022;17(2):e0263827. doi: 10.1371/journal.pone.0263827 35171949 PMC8849444

[18] Pérez YM, Gama A, Pedro AR, de Carvalho MJL, Guerreiro AE, Duarte V, et al. The links of stress, substance use and socio-demographic factors with domestic violence during the Covid-19 pandemic in Portugal. J Public Health Oxf Engl. 2022 Mar 21;fdac024. 10.1093/pubmed/fdac024 35312006

[19] O’Hara CA, Tan RKJ. Intimate partner violence before and during the COVID-19 lockdown: findings from a cross-sectional study in Singapore. Sex Health. 2022 Jun;19(3):192–201. doi: 10.1071/SH21229 35636747

[20] Campbell L, Tan RKJ, Uhlich M, Francis JM, Mark K, Miall N, et al. Intimate Partner Violence Prior to and During COVID-19 Measures in 30 Countries: A Global Cross-Sectional Study From the I-SHARE Consortium. SSRN Electron J [Internet]. 2021 [cited 2022 Dec 25]; Available from: https://www.ssrn.com/abstract=3974550

[21] Akel M, Berro J, Rahme C, Haddad C, Obeid S, Hallit S. Violence Against Women During COVID-19 Pandemic. J Interpers Violence. 2022 Jul 1;37(13–14):NP12284–309. doi: 10.1177/0886260521997953 33685271

[22] Abu-Elenin MM, Elshora AA, Sadaka MS, Abdeldaim DE. Domestic violence against married women during the COVID-19 pandemic in Egypt. BMC Womens Health. 2022 Mar 27;22(1):94. doi: 10.1186/s12905-022-01674-5 35346160 PMC8959807

[23] Moawad AM, El Desouky ED, Salem MR, Elhawary AS, Hussein SM, Hassan FM. Violence and sociodemographic related factors among a sample of Egyptian women during the COVID-19 pandemic. Egypt J Forensic Sci. 2021;11(1):29. doi: 10.1186/s41935-021-00243-5 34691785 PMC8520827

[24] Romito P, Pellegrini M, Saurel-Cubizolles MJ. Intimate Partner Violence Against Women During the COVID-19 Lockdown in Italy: A Multicenter Survey Involving Anti-Violence Centers. Violence Women. 2022 Jul;28(9):2186–203. doi: 10.1177/10778012221079374 35481785 PMC9051993

[25] Drieskens S, Braekman E, Ridder KD, Gisle L, Charafeddine R, Hermans L, et al. Domestic violence during the COVID-19 confinement: do victims feel more socially isolated? Arch Public Health Arch Belg Sante Publique. 2022 Jan 25;80(1):39. doi: 10.1186/s13690-021-00765-3 35078519 PMC8787181

[26] Dias NG, Costa D, Soares J, Hatzidimitriadou E, Ioannidi-Kapolou E, Lindert J, et al. Social support and the intimate partner violence victimization among adults from six European countries. Fam Pract. 2019 Mar 20;36(2):117–24. doi: 10.1093/fampra/cmy042 29788243

[27] Nagashima-Hayashi M, Durrance-Bagale A, Marzouk M, Ung M, Lam ST, Neo P, et al. Gender-Based Violence in the Asia-Pacific Region during COVID-19: A Hidden Pandemic behind Closed Doors. Int J Environ Res Public Health. 2022 Feb 16;19(4):2239. doi: 10.3390/ijerph19042239 35206424 PMC8871686

[28] Ojeahere MI, Kumswa SK, Adiukwu F, Plang JP, Taiwo YF. Intimate Partner Violence and its Mental Health Implications Amid COVID-19 Lockdown: Findings Among Nigerian Couples. J Interpers Violence. 2022 Sep 1;37(17–18):NP15434–54. doi: 10.1177/08862605211015213 33993788

[29] Ebert C, Steinert JI. Prevalence and risk factors of violence against women and children during COVID-19, Germany. Bull World Health Organ. 2021 Jun 1;99(6):429–38. doi: 10.2471/BLT.20.270983 34108753 PMC8164185

[30] Plášilová L, Hůla M, Krejčová L, Klapilová K. The COVID-19 Pandemic and Intimate Partner Violence against Women in the Czech Republic: Incidence and Associated Factors. Int J Environ Res Public Health. 2021 Oct 6;18(19):10502. doi: 10.3390/ijerph181910502 34639802 PMC8508297

[31] Parrott DJ, Halmos MB, Stappenbeck CA, Moino K. Intimate Partner Aggression During the COVID-19 Pandemic: Associations With Stress and Heavy Drinking. Psychol Violence. 2022 Mar;12(2):95–103. doi: 10.1037/vio0000395 35310779 PMC8932678

[32] Sun S, Sun X, Wei C, Shi L, Zhang Y, Operario D, et al. Domestic Violence Victimization Among Men Who Have Sex with Men in China During the COVID-19 Lockdown. J Interpers Violence. 2022 Dec;37(23–24):NP22135–50. doi: 10.1177/08862605211072149 35044888 PMC9502019

[33] Lyons M, Brewer G. Experiences of Intimate Partner Violence during Lockdown and the COVID-19 Pandemic. J Fam Violence. 2022;37(6):969–77. doi: 10.1007/s10896-021-00260-x 33654343 PMC7908951

[34] Abrahams Z, Boisits S, Schneider M, Prince M, Lund C. The relationship between common mental disorders (CMDs), food insecurity and domestic violence in pregnant women during the COVID-19 lockdown in Cape Town, South Africa. Soc Psychiatry Psychiatr Epidemiol. 2022 Jan;57(1):37–46. doi: 10.1007/s00127-021-02140-7 34282488 PMC8288830

[35] El-Nimr NA, Mamdouh HM, Ramadan A, El Saeh HM, Shata ZN. Intimate partner violence among Arab women before and during the COVID-19 lockdown. J Egypt Public Health Assoc. 2021 Jun 16;96(1):15. doi: 10.1186/s42506-021-00077-y 34132902 PMC8206903

[36] Yılmaz Karaman İG, Akı Z, Çanakçı ME, Altınöz AE, Özakın E. Violence Against Women During COVID-19 Pandemic: A Comparative Study from a Turkish Emergency Department. Prehospital Disaster Med. 2022 Aug;37(4):462–7. doi: 10.1017/S1049023X22000826 35587051 PMC9203417

[37] Michielsen K, Larrson EC, Kågesten A, Erausquin JT, Griffin S, Van de Velde S, et al. International Sexual Health And REproductive health (I-SHARE) survey during COVID-19: study protocol for online national surveys and global comparative analyses. Sex Transm Infect. 2021 Mar;97(2):88–92. doi: 10.1136/sextrans-2020-054664 33082232

[38] Wickham R. Secondary Analysis Research. J Adv Pr Oncol. 2019 Jun;10(4):395–400. doi: 10.6004/jadpro.2019.10.4.7 33343987 PMC7520737

[39] Hale T, Angrist N, Goldszmidt R, Kira B, Petherick A, Phillips T, et al. A global panel database of pandemic policies (Oxford COVID-19 Government Response Tracker). Nat Hum Behav. 2021 Apr;5(4):529–38. doi: 10.1038/s41562-021-01079-8 33686204

[40] World Health Organization. Researching violence against women: practical guidelines for researchers and activists [Internet]. World Health Organization; 2005 [cited 2022 Dec 25]. Available from: https://apps.who.int/iris/handle/10665/42966

[41] Schraiber LB, Latorre M do RDO, França I, Segri NJ, D’Oliveira AFPL. Validity of the WHO VAW study instrument for estimating gender-based violence against women. Rev Saude Publica. 2010 Aug;44(4):658–66. doi: 10.1590/s0034-89102010000400009 20676557

[42] Nybergh Lotta, Taft Charles, Krantz Gunilla. Psychometric properties of the WHO Violence Against Women instrument in a female population-based sample in Sweden: a cross-sectional survey. BMJ Open. 2013 Jan 1;3(5):e002053. doi: 10.1136/bmjopen-2012-002053 23793692 PMC3664346

[43] Hossain M, Heise L. Heise L, Hossain M., STRIVE Technical Brief: Measuring Intimate Partner Violence; London School of Hygiene and Tropical Medicine, London, UK; 2017. 2017.

[44] Greenland S, Daniel R, Pearce N. Outcome modelling strategies in epidemiology: traditional methods and basic alternatives. Int J Epidemiol. 2016 Apr 1;45(2):565–75. doi: 10.1093/ije/dyw040 27097747 PMC4864881

[45] Sullivan T, McPartland T, Armeli S, Jaquier Erard V, Tennen H. Is it the Exception or the Rule? Daily Co-Occurrence of Physical, Sexual, and Psychological Partner Violence in a 90-Day Study of Substance-Using, Community Women. Psychol Violence. 2012 Apr 1;2. 10.1037/a0027106 PMC3859524 24349863

[46] Salis K, Salwen-Deremer J, O’Leary KD. The Predictive Utility of Psychological Aggression for Intimate Partner Violence. Partn Abuse. 2014 Jan 1;5:83–97.

[47] Dokkedahl S, Kristensen TR, Murphy S, Elklit A. The complex trauma of psychological violence: cross-sectional findings from a Cohort of four Danish Women Shelters. Eur J Psychotraumatology. 2021;12(1):1863580. doi: 10.1080/20008198.2020.1863580 34992746 PMC8725710

[48] Buchbinder E, Band-Winterstein T. “Like a Wounded Bird”: Older Battered Women’s Life Experiences with Intimate Violence. J Elder Abuse Negl. 2003 Feb 1;15:23–44.

[49] Beaulaurier RL, Seff LR, Newman FL, Dunlop B. External Barriers to Help Seeking for Older Women Who Experience Intimate Partner Violence. J Fam Violence. 2007 Nov 1;22(8):747–55.

[50] McGarry J, Simpson C, Mansour M. How domestic abuse affects the wellbeing of older women. Nurs Older People. 2010 Jun;22(5):33–7. doi: 10.7748/nop2010.06.22.5.33.c7795 20617716

[51] Mears J. Survival is not Enough: Violence Against Older Women in Australia. Violence Women. 2003 Dec 1;9(12):1478–89.

[52] Williams EE, Arant KR, Leifer VP, Balcom MC, Levy-Carrick NC, Lewis-O’Connor A, et al. Provider perspectives on the provision of safe, equitable, trauma-informed care for intimate partner violence survivors during the COVID-19 pandemic: a qualitative study. BMC Womens Health. 2021 Aug 27;21(1):315. doi: 10.1186/s12905-021-01460-9 34452616 PMC8393774

[53] Menculini G, Tortorella A, Albert U, Carmassi C, Carrà G, Cirulli F, et al. Access to Mental Health Care during the First Wave of the COVID-19 Pandemic in Italy: Results from the COMET Multicentric Study. Brain Sci. 2021 Oct 26;11(11):1413. doi: 10.3390/brainsci11111413 34827412 PMC8615495

[54] Mubarak F, Suomi R. Elderly Forgotten? Digital Exclusion in the Information Age and the Rising Grey Digital Divide. Inq J Med Care Organ Provis Financ. 2022;59:469580221096272. doi: 10.1177/00469580221096272 35471138 PMC9052810

[55] Hague G, Thiara R, Mullender A. Disabled Women, Domestic Violence and Social Care: The Risk of Isolation, Vulnerability and Neglect. Br J Soc Work—BRIT J SOC WORK. 2010 Jan 20;40.

[56] Xavier Hall CD, Evans DP. Social comorbidities? A qualitative study mapping syndemic theory onto gender-based violence and co-occurring social phenomena among Brazilian women. BMC Public Health. 2020 Aug 18;20(1):1260. doi: 10.1186/s12889-020-09352-7 32811465 PMC7437066

[57] Kotwal AA, Holt-Lunstad J, Newmark RL, Cenzer I, Smith AK, Covinsky KE, et al. Social Isolation and Loneliness Among San Francisco Bay Area Older Adults During the COVID-19 Shelter-in-Place Orders. J Am Geriatr Soc. 2021 Jan;69(1):20–9. doi: 10.1111/jgs.16865 32965024 PMC7536935

[58] Diamond-Smith N, Conroy AA, Tsai AC, Nekkanti M, Weiser SD. Food insecurity and intimate partner violence among married women in Nepal. J Glob Health. 2019 Jun;9(1):010412. doi: 10.7189/jogh.09.010412 30774941 PMC6359930

[59] Hatcher AM, Neilands TB, Rebombo D, Weiser SD, Christofides NJ. Food insecurity and men’s perpetration of partner violence in a longitudinal cohort in South Africa. BMJ Nutr Prev Health. 2022;5(1):36–43. doi: 10.1136/bmjnph-2021-000288 35814730 PMC9237862

[60] Fedina L, Ashwell L, Bright C, Backes B, Newman M, Hafner S, et al. Racial and Gender Inequalities in Food, Housing, and Healthcare Insecurity Associated with Intimate Partner and Sexual Violence. J Interpers Violence. 2022 Dec;37(23–24):NP23202–21. doi: 10.1177/08862605221077231 35404722

[61] Fox GL, Benson ML, DeMaris AA, Van Wyk J. Economic Distress and Intimate Violence: Testing Family Stress and Resources Theories. J Marriage Fam. 2002;64(3):793–807.

[62] Mendez-Lopez A, Stuckler D, McKee M, Semenza JC, Lazarus JV. The mental health crisis during the COVID-19 pandemic in older adults and the role of physical distancing interventions and social protection measures in 26 European countries. SSM—Popul Health. 2022 Mar 1;17:101017. doi: 10.1016/j.ssmph.2021.101017 34977323 PMC8713431

[63] Gentilini U, Almenfi M, Orton I, Dale P. Social Protection and Jobs Responses to COVID-19: A Real-Time Review of Country Measures [Internet]. Washington, DC: World Bank; 2020 Apr [cited 2023 Feb 5]. Available from: https://openknowledge.worldbank.org/handle/10986/33635

