# Supplementary material for: Intimate partner violence and its correlates in middle-aged and older adults during the COVID-19 pandemic: A multi-country secondary analysis
Source: PLOS Glob Public Health. 2024 May 16;4(5):e0002500. doi: 10.1371/journal.pgph.0002500 (PMC11098409; doi:10.1371/journal.pgph.0002500)
Supplement: S1 Fig — (DOCX) [file pgph.0002500.s002.docx]

**S3 Fig: Descriptive analysis population (N=2867) and bivariate and final model population (N=1730) selection parameters from I-SHARE 2020-21.**

| 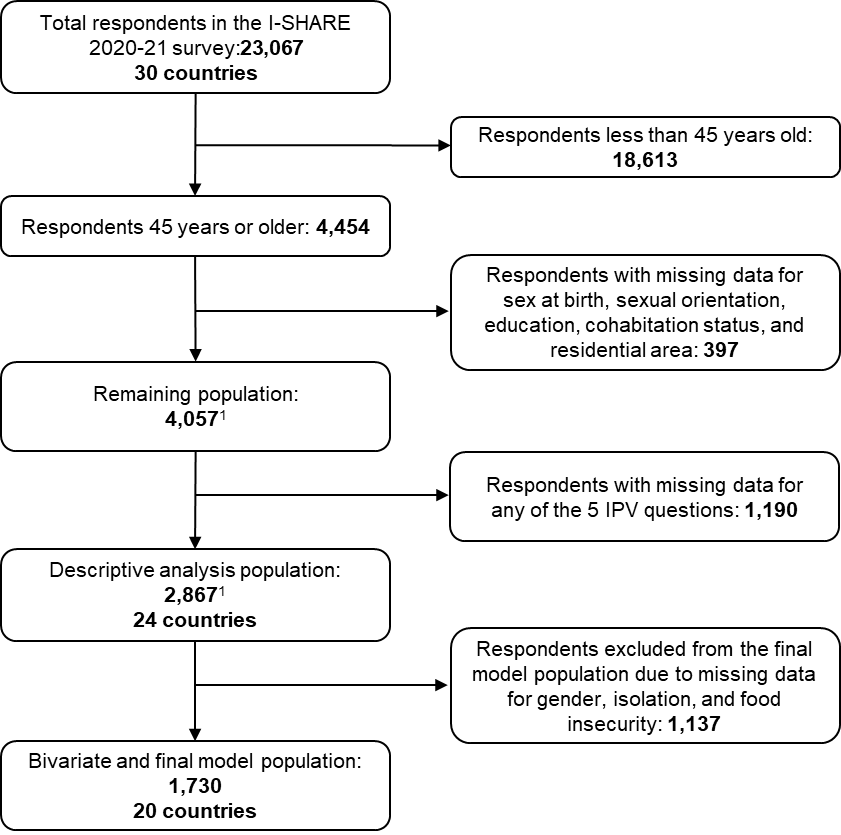  ^1^ Missing data was analysed from these populations. The characteristics of people who had missing data on the outcome and those who were dropped from the final model are described in Supplement 6. |
| --- |
