# Supplementary material for: Intimate partner violence and its correlates in middle-aged and older adults during the COVID-19 pandemic: A multi-country secondary analysis
Source: PLOS Glob Public Health. 2024 May 16;4(5):e0002500. doi: 10.1371/journal.pgph.0002500 (PMC11098409; doi:10.1371/journal.pgph.0002500)
Supplement: S1 Table — Low- (<$1,085) or lower-middle income ($1,086–4,255) economies were Nigeria, Lebanon, and Mozambique. Upper-middle income ($4,256–13,205) economies were Argentina, Botswana, Colombia, Mexico, Moldova, and Malaysia. High-income (>$13,205) economies were Australia, Canada, Czech Republic, Denmark, France, Germany, Italy, Latvia, Luxembourg, Panama, Portugal, Singapore, Spain, Uruguay, and the United States. (DOCX) [file pgph.0002500.s004.docx]

**S1 Table: Number and percentage of countries in I-SHARE 2020-21 by sampling strategy.**

Number and percentage of countries in I-SHARE 2020-21 by sampling strategy.

Low- (<$1,085) or lower-middle income ($1,086-4,255) economies were Nigeria, Lebanon, and Mozambique. Upper-middle income ($4,256-13,205) economies were Argentina, Botswana, Colombia, Mexico, Moldova, and Malaysia. High-income (>$13,205) economies were Australia, Canada, Czech Republic, Denmark, France, Germany, Italy, Latvia, Luxembourg, Panama, Portugal, Singapore, Spain, Uruguay, and the United States.

| Sampling Method | Countries | Total N | N (%) in descriptive population |
| --- | --- | --- | --- |
| Convenience Sampling | Australia | 561 | 70 (2.4) |
|  | Canada | 163 | 29 (1.0) |
|  | Colombia | 2452 | 194 (6.8) |
|  | Czech Republic | 662 | 62 (2.2) |
|  | France | 1593 | 125 (4.4) |
|  | Germany | 612 | 20 (0.7) |
|  | Italy | 329 | 33 (1.2) |
|  | Latvia | 1176 | 108 (3.8) |
|  | Luxembourg | 568 | 98 (3.42) |
|  | Malaysia | 499 | 45 (1.57) |
|  | Mexico | 1673 | 336 (11.7) |
|  | Moldova | 244 | 23 (0.8) |
|  | Mozambique | 66 | 6 (0.2) |
|  | Nigeria | 231 | 6 (0.2) |
|  | Panama | 960 | 82 (2.9) |
|  | Portugal | 3323 | 333 (11.6) |
|  | Singapore | 566 | 34 (1.2) |
|  | Spain | 295 | 54 (1.9) |
|  | Uruguay | 696 | 105 (3.7) |
|  | United States | 305 | 48 (1.7) |
|  | Total | 16974 | 1811 (0.1) |
| Representative Sampling | Czech Republic | 1200 | 464 (16.2) |
|  | Denmark | 1001 | 453 (15.8) |
|  | Total | 2201 | 917 (0.4) |
| Online Panel | Argentina | 845 | 134 (4.7) |
|  | Botswana | 344 | 3 (0.10) |
|  | Lebanon | 54 | 2 (0.1) |
|  | Total | 1243 | 139 (0.1) |
| **Total** |  | **20418** | **2867(0.1)** |
