# Supplementary material for: Intimate partner violence and its correlates in middle-aged and older adults during the COVID-19 pandemic: A multi-country secondary analysis
Source: PLOS Glob Public Health. 2024 May 16;4(5):e0002500. doi: 10.1371/journal.pgph.0002500 (PMC11098409; doi:10.1371/journal.pgph.0002500)
Supplement: S2 Table — (DOCX) [file pgph.0002500.s005.docx]

**S2 Table: I-SHARE 2020-21 adaptations of the WHO Violence Against Women Instrument (VAWI) questions used to construct IPV.**

| I-SHARE Question | I-SHARE answer options | WHO Violence Against Women Instrument Question | WHO VAW subsequent questions & answer options |
| --- | --- | --- | --- |
| Has a partner tried to restrict (online or phone) contact with your family? | No  Yes, once  Yes, multiple times | Thinking about your (current or most recent) husband / partner, would you say it is generally true that he tries to restrict contact with your family of birth? | Yes No Don't know |
| Has a partner insulted you or made you feel bad about yourself? | Same as above | Has your current husband / partner, or any other partner ever insulted you or made you feel bad about yourself? | Yes or No. *If yes continue.*  Did it happen in the past 12 months? Yes or no  In the past 12 months would you say that this has happened once, a few times, or many times?  One, few, or many  Before the past 12 month would you say that this has happened once, a few times, or many times?  One, few, or many |
| Has a partner slapped, pushed, hit, kicked or choked you or thrown something at you that could hurt you? | Same as above | Has he or any other partner ever slapped you or thrown something at you that could hurt you? | Same as above |
| Has a partner physically forced you to have sexual intercourse when you did not want to? | Same as above | Did your current husband/partner or any other partner ever physically force you to have sexual intercourse when you did not want to? | Same as above |
| Has a partner made you have sexual intercourse when you did not want to because you were afraid of what your partner might do? | Same as above | Did you ever have sexual intercourse you did not want to because you were afraid of what your partner or any other partner might do? | Same as above |
| Has a partner ever not provided money to run the house or look after the children, but has money for other things? | Same as above | NA | NA |
