# Supplementary material for: Intimate partner violence and its correlates in middle-aged and older adults during the COVID-19 pandemic: A multi-country secondary analysis
Source: PLOS Glob Public Health. 2024 May 16;4(5):e0002500. doi: 10.1371/journal.pgph.0002500 (PMC11098409; doi:10.1371/journal.pgph.0002500)
Supplement: S3 Table — (DOCX) [file pgph.0002500.s006.docx]

**S3 Table: Questions on the I-SHARE 2020-21 survey used for the analysis.**

| **Variable name** | **Question** | **Answer options** |
| --- | --- | --- |
| Age (years) | How old are you? | Free text |
| Sex at birth | Sex assigned at birth on original birth certificate | Male  Female  Other |
| Gender | How do you describe yourself? | Cisgender  Non-cisgender  Other |
| Sexual orientation | What is your sexual orientation? | Heterosexual  Bisexual  Gay  Lesbian  Questioning or unsure  Asexual  Pansexual  Other |
| Ethnic minority status | What is your ethnicity, origin group or caste? | Free text |
| Education level | What is your highest degree of schooling? | No formal education  Some primary education  Completed primary education  Some secondary education  Completed secondary education  Some college or university  Completed college or university  Other |
| Religious status | What is your religion? | Christian  Muslim  Buddhist  Hindu  Jewish  No religion  Other |
| Employment status | What was your employment status the month before the COVID-19 social distancing measures? | Employed and received a salary  Self-employed or business owner  Unemployed  Informal or piecemeal work  Retired or pensioned  Student  Other |
| Residential area | What best describes the area where you live? | (semi-)rural  (semi-)urban  Other |
| Continued on next page | | |
| Supplement 5 continued | |  |
| **Variable name** | **Question** | **Answer options** |
| Age (years) | How old are you? | Free text |
| Cohabitation status | What best describes your relationship status? | Single and never had a partner  Single but had a partner previously or currently  In a relationship but not living together  Not legally married but living with a partner  Legally married and living together  Legally married and not living together  Legally married but separated  Widowed  Divorced  Other |
| Ever isolated due to COVID-19 | Were you ever in (self-)isolation because of symptoms or because you were in close contact with someone with COVID-19 or because you returned from a country that had a large number of cases? | No  Yes |
| Food insecurity due to COVID-19 | During the COVID-measures, did you worry that your household would not have enough food? | No  Yes but less than before  Yes but no more than before  Yes more than before |
