# Supplementary material for: Intimate partner violence and its correlates in middle-aged and older adults during the COVID-19 pandemic: A multi-country secondary analysis
Source: PLOS Glob Public Health. 2024 May 16;4(5):e0002500. doi: 10.1371/journal.pgph.0002500 (PMC11098409; doi:10.1371/journal.pgph.0002500)
Supplement: S4 Table — (DOCX) [file pgph.0002500.s007.docx]

**S4 Table: Characteristics of people missing and not missing IPV outcome responses in the study from I-SHARE 2020-21 (N=4057).**

|  |  | Missing IPV outcome N (%) | Not missing IPV outcome N (%) |
| --- | --- | --- | --- |
| Age (years) | 45-54 | 567 (47.7) | 1539 (53.7) |
|  | 55-64 | 374 (31.4) | 824 (28.7) |
|  | $\geq$65 | 249 (20.9) | 504(17.6) |
| Sex | Male | 408 (34.3) | 1278 (44.6) |
|  | Female | 779 (65.5) | 1589 (55.4) |
|  | Other^1^ | 3 (0.3) | 0 |
| Education level | No formal and primary | 62 (5.2) | 70 (2.4) |
|  | Secondary | 335 (28.2) | 755 (26.3) |
|  | College/University | 702 (59.0) | 1826 (63.7) |
|  | Other^1^ | 91 (7.7) | 216 (7.5) |
| Employment status | Employed | 847 (71.2) | 2154 (75.1) |
|  | Unemployed | 47 (4.0) | 83 (2.9) |
|  | Retired | 245 (20.6) | 526 (18.4) |
|  | Other^1^ | 51 (4.3) | 104 (3.6) |
| Residential area | Rural | 372 (31.3) | 944 (32.9) |
|  | Urban | 818 (68.7) | 1923 (67.1) |
| ^1^ ”Other” was a survey response option. Participants were unable to specify further. | | | |
