# Supplementary material for: Intimate partner violence and its correlates in middle-aged and older adults during the COVID-19 pandemic: A multi-country secondary analysis
Source: PLOS Glob Public Health. 2024 May 16;4(5):e0002500. doi: 10.1371/journal.pgph.0002500 (PMC11098409; doi:10.1371/journal.pgph.0002500)
Supplement: S5 Table — (DOCX) [file pgph.0002500.s008.docx]

**S5 Table: Characteristics of people excluded from the final model due to missing data on gender, isolation, and food insecurity from I-SHARE 2020-21 (N=2867).**

|  |  | Missing from final model N (%) | Included in final model N(%) |
| --- | --- | --- | --- |
| Age (years) | 45-54 | 446 (39.2) | 1093 (63.2) |
|  | 55-64 | 329 (28.9) | 495 (28.6) |
|  | $\geq$65 | 362 (31.8) | 142 (8.2) |
| Sex | Male | 571 (50.2) | 707 (40.9) |
|  | Female | 566 (49.8) | 1023 (59.1) |
|  | Other^1^ | 0 | 0 |
| Education level | No formal and primary | 24 (2.1) | 46 (2.7) |
|  | Secondary | 508 (44.7) | 247 (14.3) |
|  | College/University | 528 (46.4) | 1298 (75.0) |
|  | Other^1^ | 77 (6.8) | 139 (8.0) |
| Employment status | Employed | 735 (64.6) | 1419 (82.0) |
|  | Unemployed | 15 (1.3) | 68 (3.9) |
|  | Retired | 370 (32.5) | 156 (9.0) |
|  | Other^1^ | 17 (1.5) | 87 (5.0) |
| Residential area | Rural | 617 (54.3) | 327 (18.9) |
|  | Urban | 520 (45.7) | 1403 (81.1) |
| ^1^ ”Other” was a survey response option. Participants were unable to specify further. | | | |
