# Supplementary material for: Intimate partner violence and its correlates in middle-aged and older adults during the COVID-19 pandemic: A multi-country secondary analysis
Source: PLOS Glob Public Health. 2024 May 16;4(5):e0002500. doi: 10.1371/journal.pgph.0002500 (PMC11098409; doi:10.1371/journal.pgph.0002500)
Supplement: S6 Table — (DOCX) [file pgph.0002500.s009.docx]

**S6 Table: A sensitivity analysis with participants from LMICs to determine if the model is sensitive within this population from the I-SHARE 2020-21 survey (N=749).**

|  |  | **aOR**^1^ **(95% CI)** | **Global P-value^2^** |
| --- | --- | --- | --- |
| Age (years) | 45-54 | 1 | 0.72 |
|  | 55-64 | 0.9 (0.5, 1.4) |  |
|  | $\geq$65 | 1.2 (0.5, 2.9) |  |
| Sex | Male | 1 | 0.67 |
|  | Female | 0.9 (0.6, 1.4) |  |
| Sexual Orientation | Heterosexual | 1 | 0.16 |
|  | Other sexual orientation | 0.6 (0.3, 1.2) |  |
| Education level | No formal and primary | 1 | 0.3 |
|  | Secondary | 0.1 (0, 2.1) |  |
|  | College/University | 0.1 (0, 1.4) |  |
|  | Other^3^ | 0.1 (0, 1.3) |  |
| Employment status | Employed | 1 | 0.09 |
|  | Unemployed | 1.2 (0.5, 3.3) |  |
|  | Retired | 1.1 (0.5, 2.8) |  |
|  | Other^3^ | 2.9 (1.3, 6.4) |  |
| Ever isolated due to COVID-19 | No | 1 | 0.02 |
|  | Yes | 1.8 (0.6, 1.7) |  |
| Cohabitation status | Not living with partner | 1 | 0.89 |
|  | Living with partner | 1 (0.6, 1.7) |  |
| Food insecurity during COVID-19 | No or less than before | 1 | 0.0004 |
|  | Yes worried more than before | 2.4 (1.5, 3.8) |  |
| Residential area | Rural | 1 | 0.08 |
|  | Urban | 2.5 (0.8, 7.3) |  |
| Country social distancing stringency level | Low | 1 | 0.02 |
|  | High | 0.5 (0.3, 0.8) |  |
| Gender inequality index | Low inequality | 1 | 0.11 |
|  | High inequality | 0.2 (0.0, 2.0) |  |
| Social progress index | Medium progressivity | 1 | 0.33 |
|  | High progressivity | 0.6 (0.2, 1.9) |  |
| World Bank country income level | Upper-middle | 1 | 0.46 |
|  | Low or Lower-middle | 1.7 (0.4, 6.9) |  |
| ^1^ Adjusted for all other variables in the table.  ^2^ Global p-values were determined by likelihood ratio tests.  ^3^ ”Other” was a survey response option. Participants were unable to specify further. | | | |
