# Supplementary material for: Intimate partner violence and its correlates in middle-aged and older adults during the COVID-19 pandemic: A multi-country secondary analysis
Source: PLOS Glob Public Health. 2024 May 16;4(5):e0002500. doi: 10.1371/journal.pgph.0002500 (PMC11098409; doi:10.1371/journal.pgph.0002500)
Supplement: S7 Table — It is defined in this analysis as physical and sexual violence using data from the I-SHARE 2020–21 survey (N = 2867). (DOCX) [file pgph.0002500.s010.docx]

**S7 Table: Sensitivity analysis of the construction of IPV. It is defined in this analysis as physical and sexual violence using data from the I-SHARE 2020-21 survey (N=2867).**

|  |  | **aOR^1^ (95% CI)** | **Global P-value^2^** |
| --- | --- | --- | --- |
| Age (years) | 45-54 | 1 | 0.01 |
|  | 55-64 | 0.4 (0.2, 0.8) |  |
|  | $\geq$65 | 1 |  |
| Sex | Male | 1 | 0.17 |
|  | Female | 0.7 (0.4, 1.2) |  |
| Sexual Orientation | Heterosexual | 1 | 0.06 |
|  | Other sexual orientation | 1.8 (1.0, 3.4) |  |
| Education level | No formal and primary | 1 | 0.07 |
|  | Secondary | 0.6 (0.2, 2.0) |  |
|  | College/University | 0.3 (0.1, 0.9) |  |
|  | Other^3^ | 0.3 (0.1, 1.3) |  |
| Employment status | Employed | 1 | 0.1 |
|  | Unemployed | 2.7 (1.1, 6.8) |  |
|  | Retired | 1.7 (0.5, 6.0 |  |
|  | Other^3^ | 2.2 (0.9, 5.2) |  |
| Ever isolated due to COVID-19 | No | 1 | 0.13 |
|  | Yes | 1.6 (0.9, 2.8) |  |
| Cohabitation status | Not living with partner | 1 | 0.59 |
|  | Living with partner | 0.9 (0.5, 1.5) |  |
| Food insecurity during COVID-19 | No or less than before | 1 | 0.02 |
|  | Yes worried more than before | 2.0 (1.2, 3.5) |  |
| Residential area | Rural | 1 | 0.61 |
|  | Urban | 1.2 (0.6, 2.6) |  |
| Country social distancing stringency level | Low | 1 | 0.06 |
|  | High | 0.5 (0.3, 1.0) |  |
| Gender inequality index | Low inequality | 1 | 0.16 |
|  | High inequality | 0.5 (0.2, 1.3) |  |
| Social progress index | Medium progressivity | 1 | 0.4 |
|  | High progressivity | 1.9 (0.5, 7.6) |  |
| World Bank country income level | High-income | 1 | 0.42 |
|  | Upper-middle | 1.8 (0.4, 7.7) |  |
|  | Low or Lower-middle | 1 |  |
| ^1^ Adjusted for all other variables in the table.  ^2^ Global p-values were determined by likelihood ratio tests.  ^3^ ”Other” was a survey response option. Participants were unable to specify further. | | | |
